# Characterization of *Apis mellifera* Gastrointestinal Microbiota and Lactic Acid Bacteria for Honeybee Protection—A Review

**DOI:** 10.3390/cells10030701

**Published:** 2021-03-22

**Authors:** Adriana Nowak, Daria Szczuka, Anna Górczyńska, Ilona Motyl, Dorota Kręgiel

**Affiliations:** 1 Department of Environmental Biotechnology, Lodz University of Technology, Wólczańska 171/173, 90-924 Łódź, Poland; dar.szczuka@gmail.com (D.S.); ilona.motyl@p.lodz.pl (I.M.); dorota.kregiel@p.lodz.pl (D.K.); 2 Faculty of Law and Administration, University of Lodz, Kopcińskiego 8/12, 90-232 Łódź, Poland; agorczynska@wpia.uni.lodz.pl

**Keywords:** *Apis mellifera*, gut microbiota, lactic acid bacteria, probiotics, pesticides, pathogens

## Abstract

Numerous honeybee (*Apis mellifera*) products, such as honey, propolis, and bee venom, are used in traditional medicine to prevent illness and promote healing. Therefore, this insect has a huge impact on humans’ way of life and the environment. While the population of *A. mellifera* is large, there is concern that widespread commercialization of beekeeping, combined with environmental pollution and the action of bee pathogens, has caused significant problems for the health of honeybee populations. One of the strategies to preserve the welfare of honeybees is to better understand and protect their natural microbiota. This paper provides a unique overview of the latest research on the features and functioning of *A. mellifera*. Honeybee microbiome analysis focuses on both the function and numerous factors affecting it. In addition, we present the characteristics of lactic acid bacteria (LAB) as an important part of the gut community and their special beneficial activities for honeybee health. The idea of probiotics for honeybees as a promising tool to improve their health is widely discussed. Knowledge of the natural gut microbiota provides an opportunity to create a broad strategy for honeybee vitality, including the development of modern probiotic preparations to use instead of conventional antibiotics, environmentally friendly biocides, and biological control agents.

## 1. Introduction

The honeybee *Apis mellifera* is a social insect species that has successfully colonized numerous ecosystems around the world and plays a crucial role in pollinating wild and cultivated plants, with substantial implications for the global economy and natural ecosystems [1]. Honeybees provide a key link in the production of food, and their economic value to the United States alone is estimated to be as much as USD 15 billion [2]. Besides their pollination value, honeybees are important because of their great agronomic and economic potential owing to the production of valuable commercial products such as wax, pollen, propolis, royal jelly, and most importantly, honey [1].

Bees are vital for the preservation of the ecosystem as they help maintain an ecological balance. They are known to have complex interactions with their environment and a diverse range of microorganisms. Understanding the relationship between honeybees and their external environment is important to maintain a hospitable environment for both humans and bees. The honeybee microbiome is central to maintaining the individual’s health, and a disrupted microbiome makes the insect susceptible to a variety of problems. Thus, research has focused on the intestinal microbiome of honeybees; its role and function in bee health, fitness, and metabolism; and its response to many physical, biological, chemical, and environmental factors [3,4,5,6,7]. Such a broad perspective is needed, considering the importance of honeybee health and the knock-on impact on environmental protection.

## 2. *Apis mellifera* Characterization

*Apis mellifera* is one of the most common floral visitors in natural environments worldwide. On average, honeybees account for 13% of floral visits across all networks. Five percent of plant species are visited by *A. mellifera* exclusively [8]. The lifespan of honeybees varies significantly depending on the moment of their emergence. Therefore, they can be classified as either short-lived summer bees or long-lived winter bees. Bees emerging in spring and midsummer live for an average of 25–40 days, while winter bees have a much longer lifespan of more than 100 days [9]. This bimodal longevity distribution presumably results from complex dynamics associated with biotic and abiotic factors, interactions between individuals in the colony, and regulatory mechanisms of individuals influenced by intracolonial conditions [10]. It has been shown to be predominantly associated with bees’ flight activity and the change in the nature of their tasks, from those performed inside the nest to the more hazardous task of foraging. This significant transition in the life cycle of an adult bee is related to both dietary and physiological changes, including a shift from a carbohydrate–protein diet to a pure carbohydrate diet [11].

The worldwide distribution of honeybees is due to the activities of beekeepers, but their native range is also large, spanning Europe, Africa, and the Middle East [12]. There are 10 species of honeybee belonging to the genus *Apis*. Phylogenetic analyses involving nuclear DNA and mitochondrial (mtDNA) markers clearly approved clustering these species into three distinct groups: Cavity-nesting bees (represented by *A. mellifera*, *A. cerana*, *A. koschevnikovi*, and *A. nulensis*), giant bees (*A. dorsata, A. laboriosa, A. dorsata binghami,* and *A. nigrocincta*), and dwarf bees (*A. florae* and *A. andreniformis*) [13]. Except for *A. mellifera*, all species are now limited to Asia, and the lineage that brought about the *A. mellifera* embodies an early split from different cavity-nesting bees, so it is thought that *A. mellifera* may have originated from Asia [12].

Honeybees live in large communities with a complex organization that depends on cooperative and altruistically motivated individuals and communication. The colony is formed by hundreds of males (drones), sterile female workers numbering between 12,000 and 90,000 depending on the season, and a single queen [14,15]. The workers are responsible for all activities that assist with reproduction: They clean combs and feed larvae; are involved in comb building, the evaporation of nectar, and guarding of the hive; and above all, they are responsible for foraging to provide the colony with food and water [14]. The duties of the queen, after nuptial flights, are limited exclusively to laying eggs. During the period of most intense development, which usually takes place at the end of spring and beginning of summer, the queen lays about 2000 eggs. Drones appear in May, and they are crucial for the reproduction process. They copulate with the queen in the air and then die. Drones that did not participate in the reproduction process are expelled from the hive at the end of July and starve to death [16,17]. The group remains consistent due to its ability to distinguish nestmates from non-nestmates, which is denoted by the presence of the guard bees at the entrance of the hive. Their function is to prevent non-nestmates from entering the nest and allow nestmates to freely move inside [15].

In the simplest terms, bee nutrition is based on nectar and pollen, the former supplying bees with carbohydrates and the latter a source of proteins, lipids, and other micronutrients. In order to obtain optimal nutrition, bees balance the intake of nutrients from these complementary food sources [18]. Adequate nutrition is crucial for the proper growth and development of a honeybee colony, while any deficits contribute to aggravation of the negative impacts of viral and fungal diseases [19]. Nutrition can be considered at three different scales, that is, in terms of colony nutrition, adult nutrition, and larval nutrition. In a colony, nutritional levels are connected by a variety of interactions between the adult bees and the brood called trophallaxis (transferring of food from one individual to another) [19]. Both larvae and adult bees are dependent on the food stores of the colony, and adult bees can adjust foraging and strategies of brood-care in accordance with the supply of the hive’s provisions [20].

Pollen is the predominant source of lipids, proteins, vitamins, and minerals. It is essential for the growth, development, and reproductive processes of honeybees [21]. It is especially important for the development of the hypopharyngeal glands and body fat in newly emerged workers, which is necessary for brood-reading and overwintering [22]. Bees collect pollen and place it on the corbiculae—structures located on the hind legs [23]. The color of the corbiculae reveals information about the flowers that were visited by bees. They most commonly appear yellow, orange, or brownish, although they can also be white, navy blue, or black. Pollen is also stored in nest cells, to which all the workers in a colony have access [24]. During pollen collection, bees display temporary specialization toward the pollen of one species. European honeybees are especially consistent in terms of the flowers on which they specialize, and their individual pollen loads usually originate from a single source. Nevertheless, at the colony level, pollen is concomitantly collected from different sources [25]. There are some plants that produce pollen that is harmful for bees. There have also been cases of poisoning of humans after ingestion of honey from poisonous plants [26]. However, poisoning occurs relatively rarely, and only when the poisonous plant is dominant in a certain area where other pollen plants are absent, and bees suffer from a lack of water. Poisoning leads to noninfectious disease of adult insects [26].

Nectar is an aqueous solution containing sugars, amino acids, organic acids, proteins fats, vitamins, and minerals. It is produced by a specialized group of cells called nectaries [27,28]. The composition of nectar is dominated by sucrose, fructose, and glucose. Honeybees are sensitive to differences in nectar composition and prefer pure sucrose over pure glucose or fructose solutions; however, in the field, nectars containing mixtures of these sugars are most commonly found [28].

Honeybees produce many different substances, namely honey, bee pollen, propolis, bee bread, royal jelly, beeswax, and bee venom, which play various functions in the life cycle of honeybees [29,30]. What makes honeybees different compared to other insects is that they hoard food. During the hoarding process, food undergoes refinement, so it differs from its original state. There are two major forms of hoarded food: honey from nectar and bee bread from pollen. They are both stored in a comb formed of wax, produced using the wax glands of adult worker bees [25].

The process of honey formation is initiated by the collection of nectar from plants. It is stored at the bottom of the esophagus in the honey stomach [31]. During transport to the hive, the nectar undergoes an enzymatic treatment. The chemical transformation is based on the hydrolysis of sucrose performed by the addition of invertase [32]. Afterward, the nectar loads are transferred from honeybee nectar collectors to food-storer bees. The food-storer bees regurgitate the nectar and deposit it into the honeycomb. The nectar then undergoes a ripening process, which consists of the further conversion of sucrose to glucose and fructose, and water evaporation [31]. The water concentration is decreased to about 17% [32]. This conversion process takes from one to three days and is finalized by the capping of the cells filled with nectar using bee wax [31].

Pollen-collecting foragers transport their pollen loads straight to cells distributed within the comb. These cells are often already packed with previous loads, which may be from different floral sources. Pollen is then processed by young hive bees that pack it tightly and add regurgitated honey, which preserves the stored pollen through its antimicrobial properties. Pollen that is packed into cells for storage is referred to as bee bread [25]. The flow of water and food in the colony of honeybees has been described in detail by Wright et al. [25].

Another bee product is royal jelly, a substance secreted in the hypopharyngeal glands of young worker bees that is used to feed the larvae of drones and worker bees during the first three days of their lives, and to feed the queen. Worker and drone larvae are fed royal jelly along with honey and pollen. Royal jelly is the only food that the adult and larvae queen bee consumes [33,34]. The most important role of royal jelly is to provide nutrition and protection for honeybee larvae during development, and it is the crucial driving force in the process of caste determination. A fertile egg becomes either a sexually perfect future queen bee that has mature ovaries for reproduction, or a sexually immature worker, which depends strictly on the dose and timing of royal jelly consumption during larval development [34]. Fed with royal jelly exclusively, queen bees are capable of developing superior features, not only in terms of physical appearance, but also strength, stamina, and longevity (queen bees can live for up to 5–7 years) [35,36]. Proteins are the major constituent of royal jelly, most of which are water-soluble, and it is because of these that the secretion exhibits antiaging, antitumoral, and insulin-like activities [37].

## 3. Honeybee Microbiota

Animals that form social communities usually employ a characteristic microbiota that is essential for various processes that occur in the body [38]. The microbiota can be defined as a complex ecosystem of microorganisms that plays a critical role in a variety of metabolic functions, including modulation of glucose and lipid homeostasis, satiety regulation, management of energy, and the production of vitamins [39,40,41]. In addition, the microbiota participates in the regulation of various biochemical and physiological mechanisms by means of the production of metabolites and other substances [42]. Furthermore, the microbiota exerts anticarcinogenetic and anti-inflammatory activities, [38] and plays a significant role in the operations of the host immune system and induction of immune responses [43]. In return, the host immune system maintains a mutualistic relationship with the microbiota. This relationship enables the induction of protective responses toward pathogens and the introduction of regulatory pathways involved in the tolerance to harmless antigens [44].

While the importance of the gut microbiota is discussed more often now, the processes responsible for the beneficial features of microbial communities remain unclear [45,46,47]. The composition of the microbial communities that inhabit the gut vary significantly between different species and within them. The diversity in composition of the gut microbiota is influenced by topographical and short-term shifts in the microbial communities, with specific microorganisms inhabiting particular niches in the host during specific growth and developmental phases of the host [48].

### 3.1. Characteristics

Insects represent the most diverse animal clade in terms of the number of species, the ecological habitats they inhabit, and their overall biomass [3]. *A. mellifera* is a useful model organism with a microbial community that displays high host adaptation. While its microbiota has some similarities with those of mammals, it has a much simpler composition. The main similarities and differences in the honeybee and human gut microbiota were reviewed previously [49].

Honeybees form huge colonies that contain thousands of nonreproductive female workers, hundreds of male drones, and only one reproductive queen [14]. Newly emerged workers have a reduced core gut microbiota or may lack it entirely [50]. Their bodies are colonized by microbial communities orally by means of social interactions with nurse bees within a few days of emergence [51,52]. During metamorphosis into pupae, the gut bacteria are excreted via defecation along with the gut epithelium, and the next colonization starts due to trophallaxis, contact with other bees, as well as from the hive [53]. The abundance of bacteria in the whole gut reaches its peak 3–5 days post-adult emergence [54]. However, taxonomic shifts take place after 3–8 days, which suggests pioneer or niche construction strains. The rectum community seems to finish the development of an emergent structure after three days. The ileum is more variable, with its final structure emerging after eight days. The most important factor influencing this process is the prevalence of core species, the host immune response related to it, and the successional alternation of the environment of ileum [4]. The workers are involved in age-associated tasks, and newly emerged bees are usually associated with hive maintenance and cleaning tasks. Therefore, the interactions with adult bees, contact with the comb, and consumption of bee bread are all potential routes of inoculation [54,55]. Dong et al. [50] analyzed the succession of *A. mellifera* workers gut microbiota from birth to senescence, i.e., from 0–40 days postemergence (dpe). The genera *Gilliamella*, *Frischella,* and *Snodgrassella* colonized the honeybee gut at 1 dpe; *Lactobacillus*, *Bifidobacterium,* and *Commensalibacter* colonized at 3 dpe, while a simultaneous reduction in *Gilliamella* was observed. At 12 dpe, significant colonization by *L. kunkeei* and *Bartonella* sp. appeared, while *Bacteroides* sp., *Escherichia* sp., *Shigella* sp., and Porphyromonadaceae decreased between 19 and 25 dpe. *Commensalibacter* sp. and *Bifidobacterium* sp. abundance was reduced at 25 dpe [50].

The microbiota of honeybees are located in different parts of the gut, including the crop (located between the esophagus and ventriculus, and used for storage and transport of nectar to the hive; also called stomach or sack); midgut; the hindgut, consisting of the ileum (a narrow tube containing six longitudinal folds) and lumen; and the distal rectum [56,57]. Only *Parasaccharibacter* sp. was found in relative abundance in worker hypopharyngeal glands [58].

It was estimated that adult workers’ guts are inhabited by characteristic, specialized microorganisms belonging to nine clusters of bacterial species [59]. Each of the clusters represents a set of bacterial strains that are related. Similar to human hosts, the microbial communities in honeybees are dominated by host-adapted species, which are highly intolerant of atmospheric oxygen; therefore, the transmission of bacterial species takes place by social interactions between hosts [60]. However, unlike mammalian gut microbiota, all of the bacterial species can be cultured in a laboratory [61].

Using 16S rDNA community surveys and metagenomics of the total DNA, it was determined that guts of worker honeybees are inhabited by nine bacterial species clusters that account for 95–99.9% of the bacteria in almost all individuals [59,62,63]. Two ubiquitous Gram-negative species—*Snodgrasella alvi* (nonfermenting sugar bacteria that form a film directly on the gut wall; family Neisseriaceae) and *Gilliamella apicola* (bacteria with the ability to ferment sugar that inhabits areas directed toward the center of the lumen; family Orbaceae)—that are members of the Proteobacteria phylum can be distinguished [2,59,63]. There are two Gram-positive species belonging to phylum Firmicutes that are ubiquitous and abundant; namely, *Lactobacillus* Firm-4 and *Lactobacillus* Firm-5, which inhabit the distal rectum [2,59]. In the majority of adult workers, *Bifidobacterium asteroides* is also found (albeit with much lower abundance) [53,61]. The mentioned bacterial species clusters are the most essential microorganisms in the honeybee gut, the so-called “core bacteria” [64]. There are also less-abundant/stable species from Proteobacteria: The Gammaproteobacteria *Frischella perrara* (Orbaceae family); the Alphaproteobacteria *Parasaccharibacter apium*, *Bombella favorum*, *Bombella mellum*, *Bombella apis* (Acetobacteraceae family, Alpha 2.2); and *Commensalibacter* sp. (Alpha 2.1) and *Bartonella apis* (Alpha 1) from the Rhizobiaceae family [50,53,59,63,65,66]. Representatives of phylum Bacteroidetes have also been identified in the honeybee gut—*Apibacter adventoris* and *Apibacter mensalis* [67,68].

**Figure 1:**
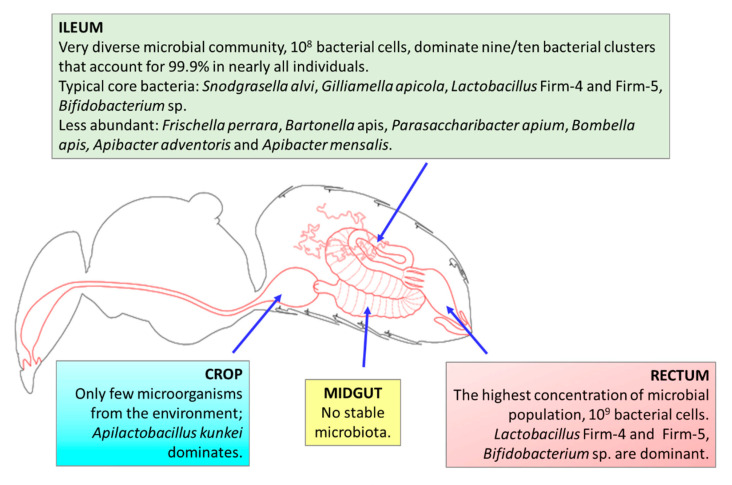
The gastrointestinal microbiota of an adult worker honeybee (*Apis mellifera*) (references in the text). Figure taken from http://honeybee.drawwing.org/book/crop (accessed on 22 March 2021) with the permission of the author.

A previous study [69] detected 10 taxa dominant in bee samples—four representatives of *Lactobacillus* sp., two *Gilliamella* sp., one *Bifidobacterium* sp., and one *Snodgrassella* sp.—that are considered to be part of the core gut microbiome of honeybees. Two of the taxa, from *Frischella* sp. and *Bartonella* sp., may vary depending on the environment. They are noncore members of honeybee gut [64]. Wang et al. [70,71] showed that the dominant phyla in honeybee GIT are Proteobacteria (63.2%), Firmicutes—(17.6%, with 15.9% of *Lactobacillus* sp.), Actinobacteria (4.1%, with 3.34% of *Bifidobacterium* sp.), and Bacteroidetes (1.7%, with 0.23% of *Bacteroides* sp.). The core member *Lactobacillus* Firm-4 was detectable in 98.4% of all analyzed bees in the study by Kešnerová et al. [64]. Tola et al. [63] analyzed *A. mellifera* gut microbiota from sub-Saharan African regions of Kenya, where indigenous and traditional management methods involving very little human intervention are practiced in beekeeping, unlike those practiced in Europe. They confirmed the core honeybee gut microbiota members were from the genera *Gilliamella*, *Snodgrassella*, *Lactobacillus* (Firm-4 and Firm-5), *Bifidobacterium*, *Frischella*, *Commensalibacter*, *Bombella*, *Apibacter*, and *Bartonella*, and that *Frischella* sp. was the third most dominant genus (16.9%), while *Lactobacillus* (Firm-4 and Firm-5) exhibited a lower abundance than has been demonstrated in other studies [63]. A summary of the GIT microbiota in honeybees is presented in Figure 1.

### 3.2. Functions

Considering an ecological perspective, gut microorganisms play a critical role in the process of codevelopment of insect-symbiotic interactions by means of secondary metabolites. Gut microbes take part in insects’ growth, development, and reproduction, and above all they contribute significantly to their metabolism [70]. Gut microorganisms synthesize essential nutritional compounds, increase the efficiency of digestion, and support insects in absorption of nutrients [72]. Most insects are inhabited by relatively few species (in comparison to mammalian gut), of which the majority is cultivable in the laboratory, but some harbor numerous communities of specialized bacteria. The factor defining limitation in gut microbiota in most insects is the lack of transmission routes between individuals. Exceptions are social insects such as termites, ants, and most importantly, bees. Social interactions give opportunities for transfer of gut microorganisms, therefore some of the most consistent and specialized gut communities, with significant functions in nutrition and protection, have been identified in social insects, such as honeybees [73].

Studies that concentrated on the beneficial health activities that microbes confer to their host have shown that the gut microbiota of honeybees plays as important a role as it does in mammals [2,3,4,45,48,49,50,72,74,75]. Two well-established functions of gut microbiota are nutrient biosynthesis and biomass deconstruction. The nutritional function was extensively studied in experiments comprising insects feeding with unbalanced and poor diets that lacked essential nutrients like amino acids and vitamins. These studies proved that insect endosymbionts help to produce nutrients that are not present in food [76]. The second function of some insect microbiota is biomass deconstruction and digestion. Both symbiotic microorganisms and host insects can release cellulolytic enzymes responsible for the deconstruction and hydrolysis of biomass, although studies have shown that microorganism activity increases the efficiency of these processes [76]. Gut microorganisms significantly contribute to the digestion of lipids and proteins, as well as the detoxification of secondary plant compounds. They also affect survival, overall size, and egg production. Moreover, they have been shown to play an important role in insect resistance against insecticides [77].

Gut microorganisms inhabiting insects can indirectly exert beneficial health effects on humans, in the case of parasitic diseases transmitted by insect vectors [78]. It was observed that in the gut of insect vectors, parasites ingested with bloodmeal reduced in number before coming into contact with host tissues. Microbial communities are thought to be an important factor influencing this effect. It was concluded that gut microorganisms contribute to the modulation of the competence of insect vectors. One of the possible mechanisms through which microbes support insects against parasites is through modification of the gut environment to constrain parasite development or induce an immune response of the host. They are also capable of producing antimicrobial peptides, which play a key role in the control of parasites and bacterial pathogens. In the study referred to above, after bloodmeal was ingested, the population of bacteria in the vector gut expanded rapidly. However, the microbiota were able to kill all parasites present [78,79]. The application of microbial symbionts to reduce vector competence is a promising approach to control the spread of insect vector transmitted pathogens [79].

Compared with the gut microorganisms of other animals, the honeybee microbiota is heavily involved in functions associated with carbohydrates, which reflects specific adaptations to a host’s diet that is rich in sugars. It provides the honeybee with sugar uptake systems belonging to various phosphotransferase systems. Many of these transporters are classified in the mannose family [73]. This feature of bacteria is important because only trace amounts of mannose are present in nectar, but it is highly poisonous when ingested at higher concentrations [73].

Another function associated with carbohydrates is enrichment of the host with arabinose efflux permeases. This family of transporters is involved in the transfer of different compounds such as antimicrobial proteins, amino acids, and sugars. A diverse set of transporters confers protection for the bacteria against a variety of pesticides applied in agriculture and naturally occurring antimicrobial proteins ingested by bees as part of their plant-based diet [3].

Furthermore, gut microorganisms influence the transformation of both nectar into honey and plant buds and exudates into propolis, owing to their fermentation properties [80]. They are also responsible for the freshness of honey [81].

One of the ways by which the gut microbiota can affect the health of honeybees is through modulation of the immune responses of the host [82]. Microorganisms impact the development and morphogenesis of the immune system and other organs and body structures [83,84]. One of the examples of how microbes affect a host is the symbiotic interaction between the fruit fly *Drosophila melanogaster* and the bacteria inhabiting its gut, *Acetobacter pomorum* [85]. This relationship influences the host’s body size, developmental rate, metabolism, activity of stem cells, and surface area of wings [85].

The primary role of gut microbiota in the functioning of mucosal immunity is not surprising, considering that the intestinal mucosa comprises the largest surface area in contact with antigens coming from the external environment, and that the dense layer of microbiota covering the mucosa constitutes the greatest proportion of antigens presented to the resident immune cells [75]. The mucosal immune system is responsible for the realization of two seemingly contradictory functions. It must tolerate microbiota inhabiting the gut to prevent the induction of harmful systemic immune responses, while controlling the number of microorganisms to avoid overgrowth and translocation [86]. Gut microorganisms are involved in the fulfillment of these objectives [75]. They control intestinal homeostasis through a variety of mechanisms involving substances like lipopolysaccharides, flagellins, and peptidoglycans. They interact with cell receptors such as Toll-like receptors, and they activate intracellular signaling pathways associated with cell survival, replication, apoptosis, and inflammatory responses [87,88,89]. In return, the host immune system controls the composition of microbes by releasing molecules like defensins, lectins, reactive oxygen species, and bacteriocins, which effectively constrain the expansion of pathogenic microorganisms [87,88,89].

Antimicrobial peptides are crucial components of innate immunity aimed at defense against the invasion of pathogens. They are determinants of the microbiota composition, as their role is to damage pathogenic microorganisms’ cells by means of membrane perforation [90]. Four families of antimicrobial peptides (abaecin, apidaecin, defensin, and hymenoptaecin) are evoked within the honeybee hemolymph during immune challenge. In one study, bees lacking gut microbiota were compared with bees inoculated with the normal gut microbiota by feeding with hive bee guts or with the bacteria *S. alvi*. It was observed that apidaecin and hymenoptaecin expression was upregulated in bees inoculated with gut microbiota, which indicates that the gut microbiota induces immune responses in bees [82].

The honeybee microbiota was observed to promote body-weight gains. To examine the effect of the microbiota on the growth of hosts, body-weight measurements were made in the presence and absence of gut microorganisms. Germ-free and conventional bees were received from pupae that were collected from hives and allowed to emerge in sterile laboratory conditions [2]. Bees deprived of microbiota achieved significantly lower weight gain (by 82%) than conventional bees. The weight gain was associated with the insulin/insulin-like signaling pathway, which plays a critical role in growth, reproduction, and aging, and regulates homeostasis and behavior in bees [2].

Gut microorganisms inhabiting insects do not just affect the digestive system. Various studies proved the existence of a gut microbiota–brain axis, meaning that gut microorganisms induce alteration of neurophysiology and changes in behavior of insect hosts [91,92]. For example, microorganisms can alter volatile profiles and the olfactory behavior of their insect hosts. Consequently, they regulate the ways in which individuals interact through chemical communication, aggregate in groups, and make decisions concerning foraging and mating. For instance, lower termite *Reticulitermes speratus* conspecific intruders are more quickly recognized and attacked when they are colonized by foreign gut bacteria releasing unfamiliar scents. Another example is found with the leaf-cutting ant *Acromyrmex echinatior*, in which suppression of the gut microbiota induces aggression among non-nestmates through alterations in cuticular hydrocarbon profiles [93]. Gut microorganisms can also increase the longevity of insects. An example of such activity of microbes is in *D. melanogaster,* the lifespan of which was significantly elongated after application of probiotic and symbiotic formulations. These formulations rescued metabolic stress markers through management of insulin resistance and energy-regulatory pathways [91]. Gut microorganisms also affect the neurophysiological development of the host, as they support cognition by enhancing its capacity to memorize and learn. A recent study linked gut microorganisms with markers of Alzheimer’s disease [93].

The gut microbiota of honeybees was observed to impact the neurophysiology and behavior of hosts. Microbes can also affect host behavior by alteration of the levels of biogenic amines such as serotonin, octopamine, and dopamine. Levels of these compounds vary seasonally in the worker’s brains, increasing in summer when foraging activity is the highest, and at different life stages, being lower in brains of newly emerged, germ-free bees [94]. Furthermore, the gut microbiome plays a key role in the regulation of social behavioral features in honeybees [95].

Gut-microbiota involvement in xenobiotic metabolism has been known for years, and this ability sheds light on the potential ability to maintain microbiota as a target for drugs to effectively contribute to treatment for various diseases [96,97]. As honeybees are exposed to a wide range of pesticides, an important role of their gut microbiota is the detoxification of xenobiotics, especially neonicotinoid insecticides [98]. Wu et al. [98] demonstrated that honeybee gut microbiota contribute to the host’s endogenous detoxification and resistance to thiacloprid and fluvalinate, as it promotes the expression of detoxification enzymes in the midgut. The importance of honeybee gut microbiota was also illustrated by a metagenome project in which symbionts of honeybees were affected by viruses. This led to detrimental effects on the growth and development of bees, and could be a major cause of colony collapse disorder (CCD) [76]. Undigested pollen was observed in the fecal content of honeybees that died due to CCD, and it indicated a deficit in the abundance of beneficial probiotic bacteria in the GIT. This may have been caused by pesticides and antibiotic residues [99].

**Figure 2:**
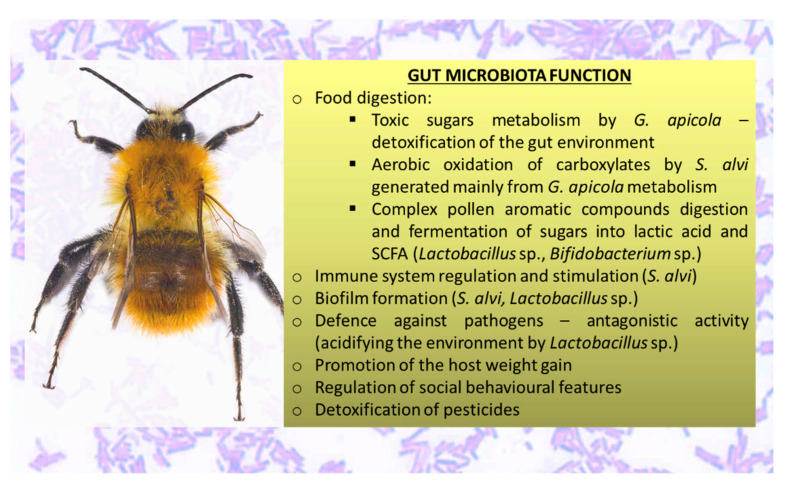
Summary of the main functions of *Apis mellifera* gut microbiota (references in the text).

The microbiota synthesizes enzymes such as proteases and glycosidases, metabolizes indigestible polysaccharides, produces essential vitamins, and conducts xenobiotic metabolism. This significantly expands the host’s biochemical capacity [100]. The fermentation of indigestible carbohydrates and oligosaccharides by bacteria belonging to the genera *Bacteroides*, *Roseburia*, *Bifidobacterium*, and *Faecalibacterium* results in the formation of short-chain fatty acids (SCFAs) including butyrate, propionate, and acetate [71,101]. These substances provide rich sources of energy for the host. Butyrate helps prevent the accumulation of toxic byproducts of metabolism [101]. Honeybee gut microbiota functions are presented in Figure 2.

### 3.3. Factors Affecting Honeybee Microbiota

Interactions between the honeybee gut community and the environment are complex and not well understood. There exists a huge diversity of gut microorganisms among insects, influenced by many factors such as habitat, feeding preference, life stage, and host species. Jones et al. [59] showed that the broad landscape influenced the diversity of some members of honeybee gastrointestinal microbiota, especially those belonging to Proteobacteria and Firmicutes. Muñoz-Colmenero et al. [102] demonstrated that the environment plays the main role in determining honeybee microbiota, and that agricultural treatments cause disruption to the bacterial community.

Many pesticides (e.g., chlorothalonil, imidacloprid, and coumaphos) contribute to important adverse health effects [7,103,104,105,106] and unfavorable changes in the structure and function of the honeybee microbiome [107]. Honeybees are exposed to them through contaminated nectar, pollen, and water. The abundance of Lactobacillales in honeybees exposed to chlorothalonil was significantly lower compared to a control group [108]. Sublethal doses of insecticides, such as fipronil, imidacloprid, thiamethoxam, and coumaphos, induced significant decreases in the quantity of *Lactobacillus* sp. and *Bifidobacterium* sp. regardless of season [108]. Exposing honeybees to glyphosate negatively affected the gut microbiome, leading to a decreased total number of gut bacteria and reduced amounts of *S. alvi, Bifidobacterium,* and *Lactobacillus* (Firm-4 and Firm-5) [52]. Motta et al. [109] investigated the effects of glyphosate on bees under laboratory and field conditions, and demonstrated that honeybees transport glyphosate to the hive, which can increase the exposure of insects to xenobiotics. Furthermore, glyphosate reduced the abundance of beneficial bacteria in the honeybee gut in a dose-dependent way [109]. According to Liu et al. [110], high and very high concentrations of thiacloprid (a neonicotinoid insecticide) led to dysbiosis in the gut microbial community of honeybees. It caused a decrease in total microbial abundance in a dose-dependent manner in three treatment groups of insects. Another neonicotinoid insecticide, nitenpyram, contributed to key alterations in the microbiota community, leading to metabolic changes and a decrease in effectiveness of the immune system [111]. Alberoni et al. [112] investigated the long-term impact of two neonicotinoids (imidacloprid and thiacloprid), on worker honeybees’ gut microbiota under open-field conditions after acute and chronic exposure. Numerous negative effects were observed in several microbial species such as *Frischella* sp., *Lactobacillus* (Firm-4 and Firm-5), and *Bifidobacterium* sp., the changes of which contributed to gut dysbiosis. The general problem with pesticides and honeybees is that pest-control methods alter the composition, diversity, and physiology of gut microbiota, and consequently affect honeybee health, especially after long-term exposure [113,114]. Furthermore, exposing honeybees to pesticides negatively impacts their gut microbiome and increases their susceptibility to infection by opportunistic pathogens [112]. To date, there has been no research on the mechanisms of detoxification of neonicotinoid insecticides by LAB (likewise probiotic) with the application of cell lines. A prerequisite for the toxic effects of a pesticide is its uptake into the body (bioavailability). Future studies should test the reduction in uptake of pesticides or their metabolites in a Caco-2 gut model (passage through the gastrointestinal epithelium) under the influence of probiotics. The toxicity of metabolites of pesticides conducted by some LAB strains is not known (summarized in Table 1), and it is not known whether these metabolites are more or less toxic than the substrate.

Honeybees exhibit a complex social network of microorganisms that can be characterized by variations according to geographic location [5,114]. For example, in *A. mellifera jemenitica*, the rural honeybee characteristic of the Kingdom of Saudi Arabia, some bacteria identified in the alimentary tract—*Citrobacter* sp., *Providencia vermicola*, *Exiguobacterium acetylicum*, and *Planomicrobium okeanokoites*—are unique to this species [115]. The core honeybee intestinal microbiota is also subjected to global seasonal variations [108]. Few studies have shown how extreme modifications impact gut microbiota dynamics during overwintering. However, seasonal changes in the honeybee microbiome in Canada were investigated by Bleau et al. [53], and they observed a decrease in the abundance of Enterobacteriaceae from September to November, while the relative abundance of Neisseriaceae increased. Subotic et al. [69] found that the honeybee microbiome changes seasonally. Another study found differences in bacterial abundance of honeybee gut community members between summer and winter months that were linked to diet [64]. The lowest diversity and highest bacterial loads were observed in winter bees (with high levels of *Bartonella* sp. and *Commensalibacter* sp.) [86]. Furthermore, diet (type of sugar used in winter forage, nutritional stressors, poor-quality diet, and propolis-rich and propolis-poor diets) has been shown to determine the profile of the dominant honeybee gut community [71,116,117]. A high-fat diet (palm oil) significantly increased the abundance of *Gilliamella* sp., while a decreased abundance of *Bartonella* sp. was observed [118]. In another study, honeybees that were subjected to feeding with “aged” pollen displayed increased mortality, a higher load of *Nosema* sp., a pathogen of fungal origin, and a significant shift in the gut microbiota composition [6].

Due to the increasing risk of CCD, attempts have been made to treat colonies using chemical methods. Antibiotics can influence the host by altering the species of gut microbiota. Daisley et al. [119] documented the deleterious effects of antibiotics on the gut microbiome, immunity, and productivity of honeybees. Several residues of antibiotics and veterinary chemotherapeutics are detected in honey, showing that honeybees are still exposed to them, despite many countries banning their usage in beekeeping [120,121]. These stressors prompt a reduction of bacterial species in the honeybee gut, weakening their immunity and increasing their susceptibility to infections [122]. In one study, honeybees underwent treatment with antibiotics, which resulted in the elimination of their microbiota. It was found that bees were more susceptible to infections by *Nosema ceranae* (a frequent honeybee pathogen) due to its negative influence on the immune system, which was illustrated by the depletion of the expression of genes that encode antimicrobial peptides [54]. Another study suggests that disturbance of gut microbiota with tetracycline decreased honeybee survival, which was associated with an elevated susceptibility to the opportunistic pathogen *Serratia* sp. [6]. Furthermore, antibiotic residues may be found later in honeybee products. Ortiz-Alvarado et al. [123] studied the effect of two commercial beekeeping antibiotics—Terramycin (oxytetracycline) and Tylan (tylosin tartrate)—on bee physiology and behavior throughout development. The results of the study showed that antibiotic treatments increased the amount of lipids and the rate of behavioral development. The timing of the antibiotic treatment affected the age of onset of behaviors, starting with cleaning, then nursing and foraging. Bees treated during the larva–pupa stages demonstrated an accelerated behavioral development and loss of lipids, while bees treated from larva to adulthood had a delay in behavioral development and loss of lipids. These effects of antibiotic treatments suggest a role of microbiota in the interaction between the fat body and brain, which is important for honeybee behavioral development. Zheng et al. [49] presented an overview of the recent research in the field of antibiotic use. Long-term antibiotic use may have impacted the diversity within human gut communities and has resulted in high frequencies of resistance determinants [124]. In the United States and other countries where beekeepers used antibiotics since the late 1940s to control or prevent larval bacterial diseases (foulbrood), antibiotic exposure has affected gut communities of honeybees [125,126,127]. This practice has resulted in high frequencies of antibiotic resistance determinants in core gut bacteria isolated from bees in the United States, in contrast to gut bacteria of honeybees from countries that do not permit the use of antibiotics in beekeeping [120,128]. In both human and honeybee gut communities, resistance determinants have been exchanged among community members through horizontal transfer [129]. In the European Union (EU), legal permission for the application of antibiotics is connected with the food safety and protection of consumers. The new European environmental strategy “The European Green Deal” [130] stresses the role of the “from farm to fork” approach, which entails designing a fair, healthy, and environmentally friendly food system. The strategic plans will need to reflect an increased level of ambition to reduce the use and risk of chemical pesticides, as well as the use of fertilizers and antibiotics. The EU needs to develop innovative ways to protect harvests from pests and disease, and to consider the potential role of new innovative techniques to improve the sustainability of the food system, while ensuring that they are safe. The most significant act that regulates food safety is Regulation No. 178/2002 [131], which includes the basic rules on food safety and established the European Food Safety Agency. European food safety is regulated by over a hundred legal acts, and Regulation No. 415/2014 [132] established the EU reference laboratory for bee health, which coordinates the methods employed in the member states for diagnosing relevant bee diseases. In reference to the veterinary medicinal products as antibiotics in the bee sectors, member states have to comply with the European rules on veterinary medical products. The definition of honey is regulated in the Directive 2001/110/EC [133]. The Commission stresses the limited availability of veterinary medicines for bees. According to Regulation (EC) No 470/2009 [134], the veterinary medicinal products intended for use in food-producing animals like bees have to be scientifically evaluated according to human food-safety requirements. Regulation (EU) No 37/2010 [135] outlined the EU Maximum Residue Limits (MRLs) for residues of pharmacologically active substances in honey. For some substances (e.g., amitraz and coumaphos), an MRL has been established, while for others the evaluation demonstrated that no MRL was required to protect food safety (e.g., flumethrin, oxalic acid, and tau fluvalinate). Products that have not been assessed as safe according to these requirements can neither be authorized nor used otherwise for food-producing animals. A new Regulation (EU) No 6/2019 [136] on veterinary medical products will come into effect on 22 January 2022. The regulation sets out rules for the sale, manufacture, import, export, supply, distribution, control, and use of veterinary medicinal products (VMPs), aiming to modernize legislation, stimulate innovation in and increase the availability of VMPs, and strengthen the EU’s campaign against antimicrobial resistance. The regulation specifies clear and fully harmonized labeling requirements, adopts a simpler system for making decisions on exceptions, and uses a risk-based approach to pharmacovigilance and controls among the key measures. It defines clear rules for organically sourced VMPs and novel therapies that also aim to encourage the development of new VMPs. It is important that the regulation strengthens the EU’s fight against antimicrobial resistance by banning the preventive use of antibiotics in groups of animals, banning the preventive use of antimicrobials via medicated feed, restricting the use of antimicrobials as a control treatment to prevent a further spread of infection, introducing a reinforced ban on the use of antimicrobials for promoting growth and increasing yield (in addition to the 2006 prohibition of using antibiotics as growth promoters in feed), including the possibility to reserve certain antimicrobials for humans only, obligating EU countries to collect data on the sale and use of antimicrobials, introducing science-based maximum limits for cross-contamination of feed with antimicrobials, and introducing various other measures to promote the responsible use of antimicrobials.

Another factor influencing honeybee gut microbiome composition is exposure to particulate-matter air pollution [137], which has been investigated for the buff-tailed bumblebee (*Bombus terrestris*) [138]. Likewise, there is scant evidence on the effects of heavy metals on honeybees [139,140].

**Figure 3:**
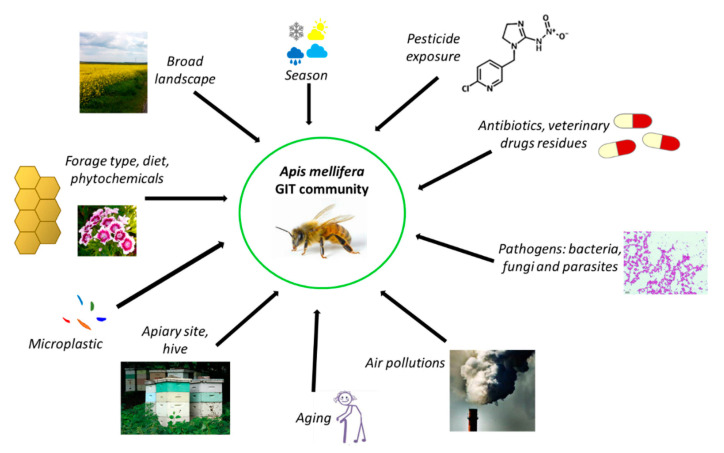
Possible factors affecting the microbiome of *A. mellifera* GIT (gastrointestinal tract) (references in the text).

In a recent study by Wang et al. [141], the authors investigated how microplastics impact honeybee fitness. They fed newly emerged bees for 14 days with microplastics under laboratory conditions. The accumulation and degradation of microplastics in the gut and interaction with gut bacteria was observed. A significant decrease in diversity and changes in the core microbial population took place. The real challenge with environmental factors affecting the honeybee microbiome, such as air pollutants, heavy metals, and microplastics, is determining the mechanism of their action and how they should be measured. Several factors influencing the honeybee gut community are presented in Figure 3.

## 4. LAB as a Significant Component of the Honeybee Microbiota and Their Beneficial Activities

Similar to other animals, in honeybees LAB are an integral part of the microbiota [142]. Microaerophilic conditions dominate the honeybee digestive system, and the temperature of 35 °C and presence of sugars from nectar are ideal conditions for lactic acid bacteria [19]. They can be characterized as Gram-positive, nonsporulating, catalase-negative bacteria that are highly tolerant to low pH [143]. These bacteria attain the shape of rods and cocci [144]. They utilize carbohydrates to obtain energy, using endogenous carbon source as final electron acceptor [145]. As the name suggests, LAB produce lactic acid [146]. Based on the products of fermentation, they can be classified either as homofermentative, producing mainly lactic acid, or heterofermentative, producing other substances such as acetic acid or ethanol [147,148]. Considering taxonomy, LAB belong to two different phyla, Firmicutes and Actinobacteria [148]. In phylum Firmicutes, LAB belong to the order *Lactobacillales*, which includes six families: Aerococcaceae, Carnobacteriaceae, Enterococcaceae, Lactobacillaceae, Leuconostocaceae, and Streptococcaceae [149]. LAB in the Actinobacteria phylum belong to the *Bifidobacterium* genus [150].

The most significant representative of LAB is *Lactobacillus* sp. This genus comprises 261 species that display extreme diversity in terms of phenotype, ecology, and genotype. Zheng et al. [151] examined the taxonomy of Lactobacillaceae and Leuconostocaceae using whole genome sequences. Their evaluation concerned parameters including core genome phylogeny, pairwise average amino acid identity, signature genes specific for clade, physiology, and ecological characteristics. They proposed to reclassify the genus *Lactobacillus* into 25 genera including an amended *Lactobacillus* genus, *Paralactobacillus,* and 23 newly introduced genera: *Acetilactobacillus, Agrilactobacillus, Amylolactobacillus, Apilactobacillus, Bombilactobacillus, Companilactobacillus, Dellaglioa, Fructilactobacillus, Furfurilactobacillus, Holzapfelia, Lacticaseibacillus, Lactiplantibacillus, Lapidilactobacillus, Latilactobacillus, Lentilactobacillus, Levilactobacillus, Ligilactobacillus, Limosilactobacillus, Liquorilactobacillus, Loigolactobacilus, Paucilactobacillus, Schleiferilactobacillus* and *Secundilactobacillus*. The description of the family Lactobacillaceae was extended to include not only genera previously belonging to the family Lactobacillaceae, but also those belonging to Leuconostocacae. This reclassification improves the understanding of the beneficial health activities of these bacteria due to the fact that species that are more closely related, and thus share more physiological features, are located in the same genus [151]. In the current text, the LAB nomenclature used follows the source references.

LAB can be found in decomposing plant materials, fermented food, sourdough, and cavities of animals, including humans [145]. These bacteria are important from a food-industry perspective because they are utilized as bioconversion agents and starter cultures in food production [152]. They are involved in the preparation of dairy products (e.g., hard cheeses, butter, yogurt, and kefir), fermented meat and fish products, and fermented vegetables (e.g., sauerkraut, pickles, and olives) [153,154,155,156,157,158,159]. They are attractive starter cultures because they produce bacteriocins, which display inhibitory activity toward food-spoilage microorganisms [152].

Various species of LAB occur in the respiratory, intestinal, and genital tracts of animals [160]. In humans, they predominantly inhabit the oral cavity, ileum, colon, and vagina [161,162]. LAB in the microbiota are involved in a variety of different functions that affect the host. For instance, LAB inhibit the expansion of pathogens in the gut as they compete for nutrients [163]. Since they are primarily fermenting saccharides, but also utilize amino acids, they can significantly deplete the nutrient resources to both saccharolytic and proteolytic species [164]. Furthermore, the products of their metabolism, such as organic acids, carbon dioxide, ethanol, or hydrogen peroxide, also contribute to the fight against pathogens [165]. LAB also produce bacteriocins—proteinaceous molecules that disturb the growth of most bacteria. They are capable of the biosynthesis of many different types of antagonistic molecules [166]. As previously described, gut microbiota are significantly involved in the immunomodulation of the host, and LAB, as a constituent of the microbiota, participate in these interactions [167]. The most prominent effect of LAB is related to the enhancement of the ratio between anti-inflammatory and proinflammatory cytokines. LAB components (e.g., lipoproteins and exopolysaccharides) may also directly induce immune responses. Furthermore, it was observed that *Lactobacillus johnsonii* induces the aggregation of *Helicobacter pylori* (a pathogen that invades the gut), which contributes to the depletion of the bacterial load and facilitates the clearance of the aggregated pathogen [168]. LAB were also observed to affect the metabolism of lipids. A study performed by Kishino et al. [168] demonstrated that *Lactobacillus plantarum* displays the ability to metabolize fatty acids and is involved in the saturation metabolism of polyunsaturated fatty acids, which leads to the generation of hydroxyl fatty acids, oxo fatty acids, conjugated fatty acids, and partially saturated *trans*-fatty acids as intermediates [168]. Fatty-acid analysis in mice revealed that intestinal microbes modify the composition of fatty acids in the host [168]. LAB were also observed to efficiently protect human and animal intestinal epithelial cells from the enteric viral infections [169]. In that study, selected LAB strains were chosen based on previous in vitro trials and were incubated with animal and human intestinal cell lines (of nontumor origin), which were further exposed to rotavirus and transmissible gastroenteric virus. It was observed that various strains displayed moderate to total cell monolayer protection against viruses. The most prominent effect was recorded for *Lactobacillus rhamnosus* and *Lactobacillus casei* Shirota. A significant antiviral effect was observed for *Enterococcus faecium*, *Lactobacillus fermentum, Lactobacillus pentosus,* and *L. plantarum* [169].

The presence of LAB within honeybees has been extensively investigated over the years. A study conducted by Vasquez et al. [57] demonstrated the presence of 13 bacterial species representing genera *Lactobacillus* and *Bifidobacterium* in the honeybee crop. Among these species, *Lactobacillus kunkei* was found to be dominant [57]. Another study performed by Olofsson and Vasquez [170] examined the microorganisms inhabiting honeybee stomach. Phylogenetic research pointed out the presence of 10 different phylotypes of LAB. Among them, five were closely related to *L. kunkeei*, *B. asteroides,* and *Bifidobacterium coryneforme*. The other five phylotypes were more distantly related, but were mostly related to the *Lactobacillus* genus [170]. Another study by Vasquez et al. [171] documented the presence of *Lactobacillus helveticus* in the honeybee stomach. Forsgren et al. [57] isolated *L. kunkeei*, *B. asteroides,* and *B. coryneforme* from the crop. Olofsson et al. [172] isolated even more strains of LAB from the honeybee crop: *Lactobacillus helsingborgensis*, *Lactobacillus kimbladii*, *Lactobacillus mellis*, *Lactobacillus mellifer*, *Lactobacillus melliventris*, *Lactobacillus apis*, *Lactobacillus kullabergensis*, *Lactobacillus apinorum*, *L. kunkeei,* and *B. coryneforme*.

The rectum, which is where fecal waste is stored prior to defecation, was also shown to be abundant in *Lactobacillus* species and *B. asteroides* clusters. *Lactobacillus* sp. can also be found in the lumen of ileum [61]. Audisio et al. [173] performed a study and examined the whole intestinal tracts of honeybees from the esophagus to the rectum. In the research, eight strains belonging to *Lactobacillus* genus and five belonging to genus *Enterococcus* were isolated. They performed 16S rRNA analysis and identified *Lactobacillus* strains that belonged to species *L. johnsonii* and *Enteroccocus* strains of *E. faecium*. Furthermore, McFrederick et al. [174] reported the presence of three other species of *Lactobacillus* in the bee gut. Based on 16S rRNA analysis and fatty-acid profiling, it was established that these strains belonged to species *Lactobacillus micheneri*, *Lactobacillus timberlakei,* and *Lactobacillus quenuiae*. In another study performed by Janashia and Alaux [175], three different LAB species were isolated from the worker honeybee gut, namely *Fructobacillus fructosus*, *Fructobacillus tropaeoli,* and *Fructobacillus pseudoficulneus*. Iorizzo et al. [19] identified 24 strains from honeybee stomach and midgut of *A. mellifera ligustica*, a native endemic Italian subspecies. Ten strains of *L. plantarum* were found in the stomach, along with three strains of *Apilactobacillus kunkeei*, one strain of *Lactococcus lactis*, and one strain of *F. fructosus*; and eight strains of *Al. kunkeei* and one strain of *L. plantarum* were found in the midgut.

Rokop et al. [176] found bacteria belonging to genera *Lactobacillus* and *Fructobacillus* in bee pollen. Janashia and Alaux [175] isolated *L. kunkeei* and *B. asteroides* from bee pollen. Anderson et al. [177] isolated bacteria belonging to genus *Lactobacillus*, which were predominantly *L. kunkeei*. Bulgasem et al. [178] examined 15 types of this bee product from different sources. The identification procedure they performed used API 50 CH tests to prove the presence of *L. plantarum*, *Lactobacillus curvatus*, *Pediococcus acidilactici,* and *Pediococcus pentosaceus*. Aween et al. [179] conducted research using commercially available honey from Malaysia and isolated 36 strains by means of API CH 50 tests, six of which were identified as *Lactobacillus acidophilus*. Asama et al. [180] noted that bacteria belonging to the *Lactobacillus* genus were dominant among samples of honey, bee pollen, royal jelly, and the whole gut and honey stomach of bees. In whole guts of bees, *Lactobacillus insectis* was most abundant, while in bee pollen, royal jelly and honey the most abundant species was *L. kunkeei*. Libonatti et al. [181] isolated *Weissella paramesenteroides* from bee bread. Anderson et al. [177] also observed the presence of *L. kunkeei* in a sample of bee bread. Iorizzo et al. [19] identified 21 strains in bee bread: 10 strains of *L. plantarum*, four strains of *F. fructosus*, three strains of *Al. kunkeei*, three strains of *Lactobacillus brevis*, and one strain of *L. lactis*. Neveling et al. [182] documented the presence of fructophilic LAB (those preferring D-fructose over D-glucose) in biological materials isolated from fresh flowers, beehive elements, and honeybees collected in Stellenbosch and the Durban Botanical Garden in Durban, South Africa. These isolates were identified as *L. kunkeei* and *L. brevis*.

Magnusson et al. [183] isolated LAB from different flowers: *P. pentosaceus* was isolated from clover (*Trifolium* L.); *P. pentosaceus* and *L. plantarum* from chestnut (*Castanea* Mill.); *Lactobacillus coryniformis*, *L. plantarum*, *Lactobacillus sakei*, *Pediococcus parvulus,* and *P. pentosaceus* from dandelion (*Taraxacum officinale*); and *L. plantarum* from lilac (*Syringa vulgaris*). In a study performed by Rodríguez et al. [184], LAB strains were isolated from passion fruit (*Passiflora edulis*) flowers, custard apple flowers (*Annona reticulate*) and meddler (*Mespilus germanica*) flowers gathered in Tucumar in northern Argentina. Six different strains were isolated from passion fruit flowers, namely *Enterococcus casseliflavus*, *Enterococcus gallinarum*, *Enterococcus faecalis*, *L. lactis* ssp. *lactis*, *Leuconostoc mesenteroides* ssp. *Mesenteroides,* and *Weisella cibara*. Two strains were isolated from custard apple flowers: *Enterococcus casseliflavus* and *L. brevis*. Four strains were isolated from medlar flowers: *E. casseliflavus*, *L. lactis*, *L. lactis* ssp. *lactis,* and *Leuconostoc pseudomesenteroides* [184]. In research conducted by Endo et al. [185], three strains of fructophilic LAB were isolated from flowers gathered in South Africa. The biological material for isolation comprised flowers of peony (*Paeonia suffruticosa*) and bietou (*Chrysanthemoides monilifera*). The isolates were closely related to *Lactobacillus fructivorans*, *Lactobacillus homohiochii*, *Lactobacillus lindneri,* and *Lactobacillus sanfranciscensis*. Based on 16S rRNA gene analysis, these three strains were classified as a novel strain with the proposed name *Lactobacillus florum* sp. nov. The presence of LAB in flower pollen proves that it can be found in the honeybee GIT and its environment, and indicates the transmission of microorganisms between honeybees and flower pollen grains and nectar [163,186].

LAB are involved in a variety of functions that affect honeybees. One of their profitable activities is the contribution to bee nutrition. It was suggested that bacteria belonging to genus *Bifidobacterium*, *Simonsiella,* or *Lactobacillus* are capable of the production of SCFAs such as acetic acid, which are waste products of carbohydrate metabolism [187]. Assimilation of these compounds can supplement the nutrition of bees. It is possible that SCFAs can be absorbed in the rectal wall of insects and it has been determined that the greatest amount of pollen and biomass of bacterial origin among adult honeybees is located inside the rectum [187]. Among the bee gut microbiota known to produce SCFAs, *Lactobacillus* Firm-5 is considered the main producer of succinate and pimelate, while *B. asteroides* is considered the main producer of valerate [49]. *A. mellifera* could obtain extra nutrition from these rectal bacteria during overwintering, as consumed food storage takes place within the rectum for longer periods of time [187].

LAB also exhibit colonization resistance against microbes that are potentially harmful, preventing the dysbiosis in the gut. They can influence the host by changing the composition of gut microbiota. In honeybees, LAB can protect against pathogens contributing to CCD such as *Paenibacillus larvae, Melissococcus plutonius, Serratia marcescens, Ascosphaera apis,* and *Nosema* sp. [188,189,190,191,192,193]. Iorizzo et al. [163] tested the antagonistic activity of 85 strains of *L. kunkeei* against *A. apis* DSM 31116, of which 23 displayed high inhibitory activity toward the fungus, and nine strains caused 100% inhibition. Tejerina et al. [194] observed 80% inhibition of *A. apis* in vivo after feeding honeybees with three strains of *Lactobacillus* sp. bacteria added to sugar syrup at 10^5^ CFU/mL concentration. *L. kunkeei*, *Lactobacillus crispatus,* and *L. acidophilus* showed the strongest antagonistic activity against a highly virulent bacterium, *P. larvae* [195]. In one study, honeybee larvae and adult bees were administered a mixture of four different strains of *L. kunkeei.* This resulted in reduced mortality related to infection of larvae by *P. larvae*, as well as a decrease in counts of *N. cerenae* spores in adult individuals [190]. Evans and Armstrong [125] considered the influence of gut microorganisms on infection with *P. larvae,* and reported that bacteria isolated from *A. mellifera* inhibited the growth of *P. larvae.* However, these host bacteria did not belong to stable, core gut microbial community. Despite successful laboratory studies against *P. larvae*, the application of LAB in field experiments is not always effective [196,197], but some results are promising [190,198]. The antimicrobial effect of LAB from the honeybee environment against bee pathogens were discussed in a review by Ramos et al. [199].

## 5. Probiotics for Honeybees

Due to their beneficial health effects, some LAB are considered probiotics. Probiotics are defined as live microorganisms that, if administered in adequate amounts, confer a health benefit on the host [200]. In order to identify the microorganism as a probiotic, it should fulfill a set of conditions [201]. Therefore, considering the fact that some LAB are probiotic, they are nonpathogenic, nontoxic, and achieve GRAS (Generally Recognized as Safe) status. They remain alive and active in GIT, are highly resistant to digestive enzymes and stomach acid, and have the ability to adhere to the intestinal epithelium [201,202]. Additionally, for a probiotic strain to possess the status “probiotic”, it should distinguish itself with a special feature characteristic of all LAB. For example, in the case of probiotics for honeybees, this could include immune-system stimulation; pathogen inhibition; or pesticide/xenobiotic degradation, binding, or neutralization. The supplementation of honeybees with probiotic LAB is a promising concept that could mitigate the harmful effects of pathogens and pesticides. However, there is no information regarding the molecular mechanisms of probiotics in protecting honeybees against pathogens. From the literature, data show that LAB are most effective in pesticide degradation during fermentation, which takes place in the GIT of honeybees. The protective effects of probiotics toward toxicity (cyto- and genotoxicity) of pesticides, especially neonicotinoid, have not been investigated comprehensively. A short review of LAB and pesticide interactions is presented in Table 1. The organophosphorus insecticide chlorpyrifos seems to be one of the most widely studied pesticides in relation to LAB. These studies indicate that the application of LAB in pesticide detoxification/removal is a safe and highly efficient method, both from the culture medium as well as during the fermentation of the contaminated food. Binding or biosorption is preferred to degradation, as the latter can generate toxic metabolites [203].

**Table 1:** Summarized effects of probiotics on pesticide mitigation, binding, degradation, metabolism, and toxicity in diverse systems.

Strain	Pesticide/s	Effect	Reference
Human gut microbiota plus *L. plantarum* ATCC 11095	Phoxim, chlorpyrifos, imidacloprid, thiamethoxam, emamectin benzoate, chlorpyrifos-d_10_, thiamethoxam-d_4_	Metabolism of pesticides in the colon digests. The rate of the metabolism was significantly increased in the presence of *L. plantarum*. The strain reduced the relative amounts of six pesticides by 11.40–86.51%.	[204]
282 LAB strains, *L. plantarum* RS60 and *P. acidilactici* D15 selected as the most efficient	Cypermethrin	229 LAB strains removed the pesticide by at least 81% (binding), and 56% of cypermethrin was removed within 15 min by *L. plantarum* RS60 and *P. acidilactici* D15. No metabolites were detected.	[203]
*L. plantarum* LB-1 and LB-2	Chlorpyrifos, deltamethrin	Degradation reached values of up to 96%. Metabolism of these insecticides was conducted by the esterase enzyme. Tested LAB used these compounds as carbon and energy sources.	[205]
*P. acidilactici* PA CNCM MA18/5 M	Thiamethoxam, boscalid	Tested pesticides deregulated genes involved in detoxification system (glutathione peroxidase-like 2, catalase) in honeybees. The strain abolished the harmful effects.	[193]
*Ent. faecium* E86, *L. lactis* subsp. *lactis* ATCC 11454; *L. rhamnosus* GG; *Leuconostoc lactis* ATCC 19256; *L. mesenteroides* subsp. *mesenteroides* ATCC 8293, *P. pentosaceus* ATCC 43200	Chlorpyrifos	All LAB degraded chlorpyrifos by a minimum of 80.3%. In the case of *P. pentosaceus,* complete degradation was observed (below detection limit).	[206]
*L. acidophilus,* *L. delbrueckii* subsp. *bulgaricus*, *L. plantarum, L. rhamnosus, L. casei,* *S. thermophilus*, *Bifidobacterium bifidum* used as starter cultures	Organochlorine pesticide mixture (α-HCH, HCB, γ-HCH, g-chlordane, α-chlordane)	The starters contributed to a significant reduction in pesticide level during the production of yogurt and cheese.	[207]
121 strains of *L. plantarum,* of which six with the highest activity were selected	Dimethoate, phorate, omethoate	All pesticides were degraded with different effectiveness depending on the strain—with omethoate, by up to 13%; phorate, by up to 36%; and dimethoate, by up to 27%.	[208]
*L. plantarum* ATCC 14917	Imidacloprid	LAB reduced susceptibility to infection with honeybee pathogen *S. marcescens* Db11 in an insect model of *D. melanogaster* by immune-deficiency pathway. LAB did not bind or metabolize imidacloprid.	[113]
*L. casei* WYS3	Chlorpyrifos	Viable pour culture bound 33.3–42% of exogenously added chlorpyrifos; acid-treated cells and heat-treated cells bound 32.0% and 77.2% chlorpyrifos, respectively. During rice straw silage fermentation, the reduction of chlorpyrifos was up to 72.0%.	[209]
*L. rhamnosus* GG (LGG), *L. rhamnosus* GR-1 (LGR-1)	Parathion, chlorpyrifos	Metabolism and passive binding of both pesticides by alive and heat-killed strains. Bacteria also reduced intestinal absorption of these compounds via Caco-2 Transwell model of the small intestine.	[210]
*L. casei*	Diazinon	Decrease of cytotoxicity of diazinon after treatment of HUVEC cells (human umbilical vein endothelial) with cell-free supernatant in a dose-dependent manner by nearly 51%.	[211]
*L. plantarum* BJ0021	Endosulfan	Protective effect of LAB, which reduced toxicity of endosulfan in pregnant Wistar rats by amelioration of blood and urine biochemical values, and decrease in apoptosis of liver and kidney cells.	[212]
10 LAB strains in skimmed milk (L*. plantarum,* *L. helveticus,* *L. brevis,* *L. bulgaricus,* *L. lactis, Streptococcus thermophilus*)	Chlorpyrifos, diazinon, fenitrothion, malathion, methyl parathion	Degradation of pesticides during fermentation of milk. The metabolism was conducted by LAB phosphatase enzymes. Different combinations of strains reduced the pesticide content to a greater extent than single strains.	[213]
*L. plantarum* DSMZ 20174	Pirimiphos-methyl	Degradation of pesticide with 81% effectiveness during wheat fermentation without toxic effect on growth and activity of the strain.	[214]
*L. fermentum* MTCC 903, *L. lactis* MTCC 4185	Chlorpyrifos	*L. lactis* and *L. fermentum* degraded chlorpyrifos to different metabolic end products—chlorpyrifos-oxon (in 61%) and 3,5,6-trichloro-2-pyridinol (in 70%), respectively.	[215]
*L. brevis* WCP902	Chlorpyrifos	Complete degradation of the pesticide. Authors isolated a gene (opdB) encoding an organophosphorus hydrolase enzyme (OpdB) responsible for the degradation.	[216]
*L. mesenteroides* WCP907, *L. brevis* WCP902, *L. plantarum* WCP931, *L. sakei* WCP904	Chlorpyrifos, coumaphos, diazinon, parathion, methylparathion	All compounds were utilized as the sole source of carbon and phosphorus during the fermentation of kimchi. Chlorpyrifos was degraded up to 100% within 9 days. Remaining pesticides were degraded by up to 82% within 12 days.	[217]

Currently, there are probiotic preparations for honeybees available on the market. Their application resulted in various profitable outcomes, including an increase in the number of bees in a colony, increased survival rates, and significant improvements of their overall health. The administration of these preparations contributed to the inhibition of development of various diseases, predominantly of fungal and bacterial origin, and the acidification of the environment, which prevents the growth of pathogens. Honeybees not only became more resistant toward pathogens, but also against stress factors [218,219]. At first it seems there are many commercial probiotic preparations for honeybees, but after screening the internet, there are several doubts related to their quality and scientific value. Some producers declare “*Lactobacillus lactis*” in the ingredients, but such bacteria do not exist, which can be confirmed at NCBI Taxonomy Browser (https://www.ncbi.nlm.nih.gov/Taxonomy/Browser/wwwtax.cgi, accessed on 22 March 2021). Other producers specify in the liquid product the presence of LAB and a dozen herb extracts, which are known for their antibacterial properties, so the survival and hence activity of LAB in a such product is doubtful. Some of the commercial products are described in too general a manner and do not provide information about the strain’s composition. It seems that there are few reliable probiotic supplements for honeybees, which we have detailed in Table 2.

## 6. Conclusions and Future Perspectives

*A. mellifera* is an important pollinator that strongly influences the genomic diversity of the plant community, helping to shape ecosystems. Moreover, honeybee products are used by humans in traditional, complementary, and integrative medicine. Maintaining bee colonies in a healthy state throughout the year is one of the main concerns of apiculture. The worrying phenomenon of disappearance of honeybee colonies is determined by several factors, namely environmental pollution, biocides, and bee diseases, and it should be stopped by applying synergistic strategies based on probiotic bacteria. The supplementation of the honeybee diet with proper probiotics could fortify the natural microbiota composition, which is important in maintaining metabolic homeostasis in bee intestines. Honeybee gut bacteria originate from their surrounding habitat, and their food, nectar, pollen, and water intake must be suitable to maintain honeybees in good condition. Beekeepers should readily adopt strategies into their beekeeping habits to help prevent colony collapse. Therefore, knowledge of molecular mechanisms of probiotics in protecting honeybee colonies against pathogens is important. It enables researchers to create new formulations suitable for the age of the bees and their function. The main challenge is searching for microbial strains that possess important probiotic features specific to honeybees and the construction of proper probiotic preparations with scientifically verified properties. In particular, lactic acid bacteria isolated from honeybees has beneficial effects on bee health and reduces the prevalence of pathogens.

One of the tools that could facilitate a better understanding of the interactions between honeybees, pathogens, and probiotics, and between honeybees, pesticides, and probiotics, are cell cultures. There is no research on mechanisms of detoxification of neonicotinoid insecticides by LAB (likewise probiotic) with the application of cell lines. A prerequisite for the toxic effects of a pesticide is its uptake into the body (bioavailability). Future studies should test the reduction in uptake of pesticides or their metabolites in a Caco-2 gut model (passage through the gastrointestinal epithelium) under the influence of probiotics. To date, the toxicity of metabolites of pesticides conducted by some LAB strains is unknown (summarized in Table 1), as is whether these metabolites are more/less toxic than the substrate. There is a need to develop a continuous honeybee cell line. Until recently, only one honeybee cell line had been defined; that is, the adherent AmE-711 fibroblast-type, which was isolated from undifferentiated embryonic tissues of *A. mellifera* [221]. Instead, many insect cell lines are applied in honeybee research [222].

**Figure 4:**
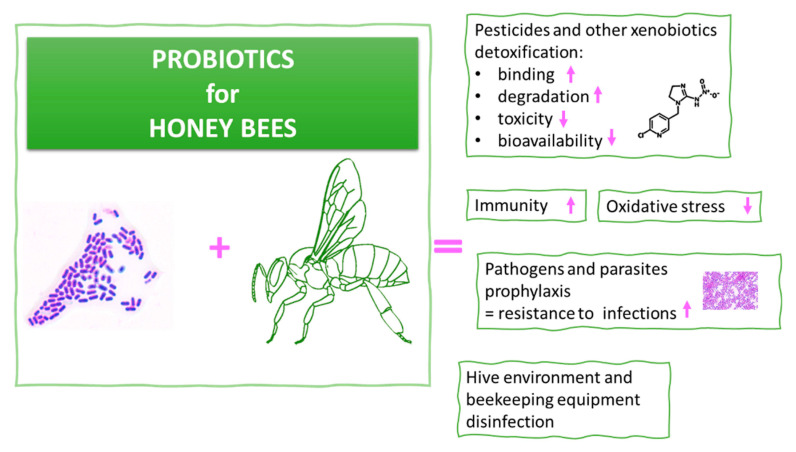
Selected challenges linked to probiotics for honeybees. The figure of the honeybee was taken from http://honeybee.drawwing.org/book/worker (accessed on 22 March 2021) with the permission of the author.

Long-term probiotic supplementation is a viable, practical, and available alternative to using chemicals and antibiotics. This option could involve natural formulations based on probiotic microorganisms, which could be applied instead of conventional antibiotics in the prophylaxis of pathogens infections, as modern biocides for hive area disinfection, and as biological control agents in plant protection. Possible future directions vary, but all strategies are interesting and beneficial to maintain healthy honeybee populations and protect the environment (Figure 4).

## Figures and Tables

**Table 2:** Short screening of probiotic honeybee supplements worldwide.

Preparation Name	Producer	Short Characteristics	Effects
Apiflora	Biowet, Poland	Lyophilized selected *Lactobacillus* strains; 1×10^8^ CFU/vial; application in water or sugar syrup. Elaborated with Maria Curie-Skłodowska University in Lublin and University of Life Sciences in Lublin, Poland.	Colonization of honeybee gut. Antagonistic effect toward *P. larvae* and *N. ceranae*. Increase of honeybee survival rate. Available at: https://biowet.pl/en/produkty/apiflora-2/, accessed on 22 March 2021
EM^®^ PROBIOTIC FOR BEES	EMRO, Japan	Multiple species of lactic acid bacteria, yeast, and photosynthetic bacteria. No detailed information given.	Inhibition of nosemosis: reduction of spore counts in colonies; colonies’ strength increased. Positive physiological changes in probiotic-treated groups of adult bees [220].
SuperDFM^®^-Honeybee	Strong Microbials, USA	Dried: *L. acidophilus, Ent. faecium, B. bifidum, L. plantarum, Saccharomyces cerevisiae, Bacillus subtilis, Bacillus licheniformis, Bacillus pumilus* fermentation products; dried *B. subtilis* fermentation extract. Total min. LAB count: 1.5 billion CFU/g. Total min. yeast count: 1 billion CFU/g.	Digestion and nutrient absorption improvement, gut health promotion, renewal of the microbes. Available at: https://www.strongmicrobials.com/honeybee, accessed on 22 March 2021
SuperDFM^®^ +P801™	Strong Microbials, USA	Composition as in the case of SuperDFM^®^-Honeybee plus *P. acidilactici* P801 fermentation product. Total min. LAB count: 2 billion CFU/g.	Strengthen and stimulate the immune system, aiding optimal nutrient absorption, better survivorship to honeybees exposed to pesticides. Available at: https://www.strongmicrobials.com/superdfm-p801, accessed on 22 March 2021
